# Editorial: Regulated cell death (RCD) in cancer: signaling pathways activated by natural products and their nano-formulations

**DOI:** 10.3389/fphar.2025.1679405

**Published:** 2025-09-02

**Authors:** Immacolata Faraone, Rocchina Miglionico, Daniela Russo

**Affiliations:** ^1^ Department of Health Sciences, University of Basilicata, Potenza, Italy; ^2^ Innovative Startup Farmis s.r.l., Potenza, Italy; ^3^ Spinoff BioActiPlant s.r.l., Potenza, Italy

**Keywords:** cancer, regulated cell death, natural compounds, nanoformulations, apoptosis resistance

## Introduction

Cancer remains one of the most significant threats to global health, characterized by high morbidity, mortality, and escalating economic costs (Filho et al., 2025). One of the most promising strategies for cancer treatment is the induction of regulated cell death (RCD), a genetically programmed and tightly controlled process involving a network of signaling pathways (Peng et al., 2022). While apoptosis is the most established and well-characterized form of RCD (Mustafa et al., 2024), increasing resistance to apoptotic signals in tumors has necessitated the exploration of alternative RCD mechanisms, such as ferroptosis, necroptosis, autophagy-dependent cell death, and paraptosis (Tong et al., 2022; Hadian and Stockwell, 2023).

The focus of current cancer research is shifting toward combination therapies that can simultaneously target multiple forms of RCD, thus reduce the likelihood of resistance and improve treatment efficacy. In this context, natural products, bioactive compounds derived from plants, fungi, and other organisms, have gained significant attention for their ability to modulate various RCD pathways. Compounds such as alkaloids, flavonoids, terpenoids, and saponins have shown strong therapeutic potential across diverse cancer types (Chen et al., 2023).

A range of recent studies have provided compelling evidence for the therapeutic efficacy of natural products in modulating regulated cell death across different cancer types.

Abusaliya et al. explored the anticancer potential of prunetrin, a glycosyloxyisoflavone, in hepatocellular carcinoma (HCC). The study demonstrated that Prunetrin significantly altered gene expression in Hep3B cells. Notably, genes associated with cell differentiation, cell cycle progression, and migration were downregulated, while those involved in exocytosis and immune activation were upregulated. The identification of three central gene targets, TP53, TGFB1, and CASP8, suggests that Prunetrin may promote apoptosis and immune-mediated cell death, positioning it as a potential candidate for liver cancer treatment.

In a complementary study, Wang et al. investigated the cytotoxic effects of metabolites derived from the fungus *Aspergillus fumigatus* YB4-17. Among these, Ent-fumiquinazoline J, a rare alkaloid, demonstrated selective toxicity against HepG2 cells. It induced both apoptosis and paraptosis, a non-apoptotic form of RCD characterized by extensive cytoplasmic vacuolation. The dual action of this compound could prove beneficial in circumventing apoptosis-resistant cancer cells, a common problem in advanced HCC therapy.

In the context of multiple myeloma (MM), Cao et al. presented a novel use for adapalene, a third-generation synthetic retinoid commonly used in dermatology. Adaptalene was shown to induce both ferroptosis and apoptosis in MM cells by downregulating GPX4, BCL-2, and SLC7A11, and upregulating cleaved caspase-3 and PARP. It also suppressed NF-κB signaling, a pathway implicated in MM progression and drug resistance. Importantly, Adaptalene restored bortezomib sensitivity in resistant cells, suggesting strong synergy and supporting its repurposing as an adjunct therapeutic agent in MM with limited toxicity to normal cells.

Wang et al. investigated paucatalinone A, a novel geranylated flavanone from *Paulownia catalpifolia* Gong Tong, for its anti-osteosarcoma activity. *In vitro*, it inhibits cell proliferation, arrests the G0/G1 cell cycle, induces apoptosis via reactive oxygen species (ROS) accumulation, calcium dysregulation, mitochondrial damage, and caspase-3 activation. Paucatalinone A disrupts cytoskeletal integrity, inhibits cell migration, and downregulates anti-apoptotic proteins (Bcl-2, Mcl-1) while suppressing ERK1/2 and cyclin D1 pathways. *In vivo*, it significantly reduces tumor growth in a murine osteosarcoma model without systemic toxicity. The results highlight its strong therapeutic potential by promoting mitochondrial-mediated apoptosis, making it a promising plant-derived candidate for osteosarcoma treatment.

Qi et al. review the anticancer mechanisms of shikonin, a valuable ingredient for the formulation of medicinal products as listed in the 2020 edition of the Chinese Pharmacopoeia, focusing on its regulation of ROS. While moderate ROS levels support cancer progression, excessive ROS can trigger cancer cell death. Shikonin exploits this by elevating ROS beyond tolerable thresholds in tumor cells, leading to apoptosis, necroptosis, autophagy, ferroptosis, and cell cycle arrest. It disrupts mitochondrial function, activates caspases, suppresses antioxidant systems like GPX4 and xCT, and inhibits survival pathways such as PI3K/Akt/mTOR. The compound also enhances sensitivity to chemotherapy in drug-resistant cancers like colorectal and lung cancer.

Min et al. demonstrated that the ginsenosides Rh2 and Rg3, two main active compounds in *Panax ginseng* C. A. Meyer, exert potent anticancer effects against non-small cell lung cancer (NSCLC) by coordinating autophagy induction and choline phosphatidylcholine metabolism reprogramming. Using immunofluorescence, Monodansylcadaverine staining, and electron microscopy, the authors show that Rh2 and Rg3 robustly induce autophagic cell death via endoplasmic reticulum stress triggered autophagy pathways. Concurrently, metabolomic profiling reveals that both ginsenosides significantly alter the choline–phosphatidylcholine metabolic axis, suggesting that disruption of lipid metabolism contributes to their cytotoxicity. This dual action—activating autophagic cell death while modulating membrane lipid metabolism, provides new insight into NSCLC suppression by ginsenosides.

Despite these promising attributes, the poor physicochemical properties of many natural compounds, such as low solubility, instability, and poor oral bioavailability, remain a major challenge. To overcome these limitations, nanotechnology has emerged as a powerful platform to enhance drug solubility, protect active ingredients from degradation, improve cellular uptake, and allow targeted delivery to tumor tissues (Andreani et al., 2024).

Pandey et al. summarized the activity of bergenin, a natural isocoumarin with broad-spectrum anticancer effects in cervical, liver, lung, and prostate cancers. Bergenin exerts its effects through ROS production, DNA damage, and mitochondrial dysfunction, while modulating several signaling pathways, including PI3K/AKT/mTOR, STAT3, and NF-κB. It upregulates pro-apoptotic proteins such as Bax and caspase-3, and downregulates the anti-apoptotic Bcl-2, thus shifting the cell toward apoptotic death. Due to its poor gastrointestinal absorption and low bioavailability, bergenin has been formulated into nanostructured lipid carriers, phospholipid complexes, and silver nanoparticles, significantly enhancing its delivery and anticancer efficacy.

## Final remarks

The research presented in this Research Topic underscores the rich potential of natural products to activate various regulated cell death pathways, offering new therapeutic angles for cancers that are resistant to conventional treatments. Furthermore, nanotechnology-based formulations address longstanding Research Topic of poor drug solubility and systemic delivery, advancing these bioactive compounds toward clinical relevance. Continued interdisciplinary efforts integrating natural product chemistry, cancer biology, and drug delivery science are essential for developing next-generation anticancer therapies rooted in nature. This integrated approach not only promises more effective and targeted treatments but also fosters innovation in overcoming drug resistance and improving patient outcomes.
